# Evaluation of urinary neutrophil gelatinase‐associated lipocalin to detect renal tubular damage in dogs with stable myxomatous mitral valve disease

**DOI:** 10.1111/jvim.16503

**Published:** 2022-10-05

**Authors:** Roberta Troia, Maria Chiara Sabetti, Serena Crosara, Cecilia Quintavalla, Giovanni Romito, Chiara Mazzoldi, Francesca Fidanzio, Maura Cescatti, Walter Bertazzolo, Massimo Giunti, Francesco Dondi

**Affiliations:** ^1^ Department of Veterinary Medical Sciences Alma Mater Studiorum—University of Bologna Bologna Italy; ^2^ Department of Veterinary Sciences University of Parma Parma Italy; ^3^ Fondazione IRET Bologna Italy; ^4^ Mylav‐Laboratorio La Vallonea Milan Italy

**Keywords:** acute kidney injury, cardiorenal syndrome, heart failure, renal biomarker, tubular damage

## Abstract

**Background:**

Dogs with myxomatous mitral valve disease (MMVD) can experience progressive renal tubular damage and dysfunction. The prevalence of renal tubular damage is not known in dogs with stable MMVD.

**Objective:**

To evaluate renal tubular damage in dogs with stable MMVD by evaluation of urinary neutrophil gelatinase‐associated lipocalin (NGAL).

**Animals:**

Ninety‐eight MMVD dogs grouped according to the American College of Veterinary Internal Medicine (ACVIM) staging (group B1, n = 23; group B2, n = 27; group C + D, n = 48) and 46 healthy dogs.

**Methods:**

Multicenter prospective observational study. Serum and urine chemistry including NGAL reported as uNGAL concentration (uNGAL) and normalized with urinary creatinine (uNGALC) were compared between MMVD dogs and healthy controls, and among different MMVD ACVIM stages.

**Results:**

The MMVD dogs had significantly higher uNGAL and uNGALC (1204 pg/mL; range, 30‐39 732 and 1816 pg/mg; range, 22‐127 693, respectively) compared to healthy dogs (584 pg/mL; range, 56‐4072 and 231 pg/mg; range, 15‐2407, respectively; *P* = .002 and *P* < .0001, respectively). Both uNGAL and uNGALC increased with the increasing ACVIM stage (*P* = .001 and *P* < .001, respectively).

**Conclusions and Clinical Importance:**

Renal tubular damage is present in dogs with stable MMVD, as measured by increased uNGAL. This tubular damage is subclinical, occurs in all stages of MMVD even in the absence of azotemia, and increases with the severity of MMVD. Reno‐protective approaches to manage MMVD dogs should be explored to slow the progression of renal tubular damage in these patients.

**Abbreviations**
ACEI: angiotensin converting enzyme inhibitor
ACVIM: American College of Veterinary Internal Medicine
AKI: acute kidney injury
CRS: cardiorenal syndrome
CHF: congestive heart failure
CKD: chronic kidney disease
CRP: C‐reactive proteins
IRIS: International Renal Interest Society Stage
MMVD: myxomatous mitral valve disease
NGAL: neutrophil gelatinase‐associated lipocalin
RAAS: renin‐angiotensin‐aldosterone system
RI: reference interval
sCr: serum creatinine
uNGAL: urinary neutrophil gelatinase‐associated lipocalin
uNGALC: urinary neutrophil gelatinase‐associated lipocalin to urinary creatinine ratio
uCr: urinary creatinine
UPC: urine protein‐to‐uCr ratio
USG: urine specific gravity
VUH: Veterinary University Hospital

## 1 INTRODUCTION

Myxomatous mitral valve disease (MMVD) is the most common acquired cardiovascular disease in dogs.^1^ The disease generally has a slow but progressive course, progressing from mitral valve insufficiency to secondary chamber dilatation and, ultimately, congestive heart failure (CHF).^1^, ^2^ Dogs with CHF require life‐long pharmacological support including diuretic treatment, and can develop progressive renal damage and dysfunction.^1^, ^3^ Indeed, the prevalence of chronic kidney disease (CKD) seems higher in dogs with MMVD compared to the general population of dogs,^4^ and azotemia increases with the severity of cardiac disease and diuretic need.^5^

Renal involvement associated with impaired cardiac function secondary to chronic heart diseases, as in the course of MMVD, is known as cardiorenal syndrome (CRS) type II.^3^, ^6^, ^7^ When MMVD is severe enough to cause CHF, persistent renal hypoperfusion, chronic congestion of the kidneys and maladaptive neurohormonal changes associated with chronic sympathetic stimulation occur and contribute to progressive renal damage.^3^, ^7^ More specifically, 2 main mechanisms of renal damage in chronic heart diseases have been proposed: intermittent acute kidney injury (AKI) episodes or sustained kidney injury occurring simultaneously with progressive reduction of functional kidney mass.^3^ Currently, serum creatinine concentration (sCr) is routinely used to monitor renal function in dogs with MMVD, although it is not a sufficient indicator of early renal damage or worsening organ function and cannot discriminate between functional and structural injury.^3^

Neutrophil gelatinase‐associated lipocalin (NGAL) is a protein belonging to the lipocalin family normally secreted in low amounts by renal tubular epithelial cells and other specific tissues.^8^, ^9^ Freely circulating NGAL is filtered through the glomeruli and almost completely reabsorbed in the proximal tubules.^10^ Urinary NGAL concentration is very low under normal physiologic conditions. In active renal tubular injury, NGAL synthesis and secretion are increased and reabsorption is decreased, resulting in increased urinary concentrations of this biomarker.^10^, ^11^ Therefore, in human and in veterinary medicine, uNGAL represents an early and sensitive biomarker of renal tubular damage.^12^, ^13^, ^14^ Specifically, it has been recognized as 1 of the earliest and most strongly induced proteins in both ischemic and nephrotoxic animal models of renal injury, and also has emerged as a promising biomarker for AKI in the clinical setting.^12^, ^13^, ^14^, ^15^

Beyond the kidney, NGAL shows potential as a biomarker in cardiovascular disease.^16^ Increased concentrations of both serum and uNGAL have been reported in many cardiovascular conditions in people, including both acute and chronic heart diseases leading to CHF.^16^ To our knowledge, an increase in serum NGAL associated with the development of renal dysfunction has been documented in dogs with acute (ie, ongoing) CHF.^17^ However, no data regarding uNGAL are available in dogs with stable compensated MMVD.

We aimed to (1) determine whether uNGAL differs between dogs with stable MMVD and healthy controls, and (2) assess uNGAL in dogs with MMVD according to the American College of Veterinary Internal Medicine (ACVIM) staging system. Our hypothesis was that uNGAL increases in dogs with MMVD if compared to healthy control dogs, and that increasing uNGAL is detected with increasing MMVD ACVIM stage.

## 2 MATERIAL AND METHODS

Ours was a multicentric prospective, observational case‐control study performed at 2 Veterinary University Hospitals (VUH; University of Bologna and University of Parma, Italy) between March 2020 and March 2021. The study was approved by the local Scientific Ethical Committee for Animal Testing (Protocol N. 747 of October 13, 2016).

### 2.1 Study population

Privately‐owned dogs were enrolled at the cardiology services of both centers. All clinical examinations and cardiac ultrasound examinations were performed or reviewed by a board‐certified cardiologist (GR or SC) at both centers. Dogs were eligible for inclusion if affected by MMVD at different ACVIM stages, diagnosed, and classified according to the current guidelines.^1^ Included dogs were in stable condition (ie, in the case of dogs in stages C and D, subjects had experienced a previous episode of CHF but were free from clinical and radiographic signs of CHF at the time of enrollment). Dogs were grouped according to ACVIM stage as follows: asymptomatic dogs without echocardiographic evidence of left‐sided cardiac remodeling were considered to be in stage B1 (group B1); asymptomatic dogs with echocardiographic signs of left‐sided cardiac remodeling (ie, left atrial‐to‐aortic root ratio ≥1.6 and body weight normalized left ventricular internal diameter in diastole ≥1.7) were considered to be in stage B2 (group B2); dogs in which at least 1 episode of CHF had occurred were considered to be in stage C; whereas dogs experiencing relapses of CHF despite regular administration of more than a total daily dosage of 8 mg/kg of furosemide or an equivalent dosage of torasemide (approximately 10% of the dose of furosemide) along with standard doses of the other medications thought to control the cardiac compromise (eg, pimobendan) were considered to be in stage D.^1^ Because of the low number of dogs in stage D, dogs in stages C and D were grouped together for statistical purposes (group C + D). A 12‐hour fast had to be enforced before the time of sample collection, but water was always available.

Exclusion criteria were the following: presence of other acquired or congenital cardiac disease, acute CHF requiring emergency treatment (eg, hospitalization and IV administration of furosemide), presence of ≥1 concomitant systemic disease including endocrinopathies, neoplasia, International Renal Interest Society Stage (IRIS) 3 and 4 CKD, acute kidney injury (AKI), acute or chronic gastroenteropathy with malabsorption, evidence of systemic inflammatory disease or sepsis, and administration of nephrotoxic drugs or other concomitant treatments (eg, cortico‐steroids, nonsteroidal antiinflammatory drugs). In contrast, disturbances of cardiac rhythm associated with MMVD did not  were not criteria for exclusion. Because urinary tract inflammation can affect uNGAL concentrations,^18^ dogs with pyuria on fresh urine sediment examination (>5 white blood cells per high power field) as well as dogs with clinical and laboratory signs of urinary tract infection or inflammation also were excluded. Cardiac treatments routinely used for MMVD, such as diuretics (eg, furosemide, torasemide), pimobendan, angiotensin converting enzyme inhibitors (ACEI; eg, benazepril, enalapril), spironolactone, and antiarrhythmic drugs were allowed.

Healthy dogs (n = 46) were included as controls for comparative purposes and to calculate the reference interval (RI) for uNGAL. These dogs were owned by medical staff or veterinary students attending the VUH. Dogs were considered healthy in the absence of any signs of illness on clinical examination and within the previous 2 months, and in the absence of clinically relevant clinicopathological abnormalities on CBC, serum biochemistry profile, and urinalysis. Dogs had not received any medications within the preceding 2 months before inclusion in the study, except for routine preventive healthcare.

### 2.2 Clinical and clinicopathological evaluation

Recorded clinical data were signalment, body weight, medical history, physical and echocardiographic examination findings, current medications, and dosage.

Blood was collected by standard venipuncture using blood vacuum collection systems; concurrent fresh urine samples were collected by spontaneous voiding or cystocentesis. Blood and urine specimens were processed according to standard procedures, and evaluated within 1 hour of collection as previously reported.^19^ When it was not possible to perform the bio‐chemistry analysis within 1 hour, samples were stored at −80°C for up to a maximum storage period of 2 months.

The biochemistry profile included sCr, urea, total protein, albumin, C‐reactive protein (CRP), and serum electrolyte (sodium, chloride, potassium, magnesium, calcium, phosphate) concentrations. Serum biochemistry was performed using an automated chemistry analyzer (AU480, Beckman Coulter, Brea, California).

Urinalysis included urine specific gravity (USG) evaluated using a hand‐held refractometer (American Optical, Buffalo, NY), dipstick tests (Combur‐Test 10 UX, Roche, Switzerland) read by an automated reader (URISYS 1100, Roche, Switzerland) and confirmed by visual inspection, microscopic sediment evaluation performed at low power field (100×) and high‐power field (400×), and urine chemistry. Urine sediment was obtained after 5‐minute centrifugation at 450*g*. Urine supernatants were immediately analyzed by dipstick examination, and then used for chemical analyses or stored. Urine chemistry was determined using the same automated chemistry analyzer used for serum biochemistry, and included urinary creatinine (uCr), total protein concentrations and urine protein‐to‐creatinine ratio (UPC).

### 2.3 Urinary NGAL evaluation

Urinary NGAL was measured using a commercial sandwich ELISA according to the manufacturer's instructions (Dog NGAL ELISA kit, BIOPORTO Diagnostics, Hellerup, Denmark) and as previously reported.^13^ Aliquots of the urine supernatant of dogs with MMVD and of control dogs enrolled in the study were stored at −80 C for up to 2 months until assayed. The assay was validated in our laboratory for dogs following a validation protocol including linearity and intraassay variation, and validation results were similar to those previously reported and consistent with those reported by the manufacturer.^20^ Urine samples from healthy dogs were diluted 1:100 whereas for dogs with MMVD an initial dilution of 1:100 was used, followed by 1:300, 1:500, and 1:900 dilutions for samples where analyte concentration could not be determined. The concentration of uNGAL in the samples was determined by measuring the absorbance of the solution at 450 nm using an appropriate plate reader (DV990BV4 spectrophotometer, N.T. Laboratory s.r.l. Calenzano, Italy) and calculating from a standard curve using curve‐fitting software (GraphPad Prism software, version 6, San Diego, California).Results were expressed as uNGAL concentrations (pg/mL) and as uNGAL‐to‐uCr ratio (uNGALC; pg/mg).

### 2.4 Statistical analysis

Data distribution was assessed using the Shapiro‐Wilk test. Data were expressed by standard descriptive statistics and presented as mean ± SD or median and range (minimum‐maximum) based on normal or nonnormal data distribution. Data obtained in healthy dogs were used to calculate the RI for uNGAL and uNGALC using the Robust method considering a right‐sided distribution. The Mann Whitney *U* test and the Student's *t*‐test were used to compare dogs with MMVD to healthy control dogs. The Kruskal‐Wallis test with post hoc comparison (Conover test) was used to compare continuous variables among dogs with MMVD of different ACVIM stage (group B1 vs B2 vs C + D). Categorical variables were compared among groups using the chi‐squared test. Spearman's correlation coefficient was used to assess potential correlations between variables. Results were considered significant if *P* < .05. Statistical analyses were performed using a commercially available statistical software package (MedCalc Statistical Software version 19.5.1; Ostend, Belgium).

## 3 RESULTS

### 3.1 Baseline characteristics

The study population consisted of 98 MMVD dogs: 23/98 (23%) in group B1, 27/98 (28%) in group B2, and 48/98 (49%) in group C + D. In group C + D, 39/48 (81%) dogs were staged in ACVIM C and 9/48 (19%) dogs in ACVIM D. Overall, 43/98 (44%) were females (17/43 spayed) and 55/98 (56%) were males (9/55 castrated); 48/98 (49%) were mixed breed dogs whereas 50/98 (51%) were purebred dogs. Median body weight was 8.9 kg (range, 2.4‐31.9) and mean age was 11 ± 2.7 years. Dogs in group B1 were significantly younger compared to those in groups B2 and C + D (*P* = .003). Body weight was significantly higher in group B1 vs group C + D (*P* = .03). Demographic data for enrolled dogs and for the different groups are reported in Table 1.

**TABLE 1:** Demographic data and descriptive statistics of the study population: dogs with myxomatous mitral valve disease (MMVD) in different ACVIM stage (group B1, group B2, group C + D), and healthy dogs

Population	Group B1 (n = 23)	Group B2 (n = 27)	Group C + D (n = 48)	Healthy dogs (n = 46)	*P* value
Age (y)	9.4 (±2.7)^b^, ^c^, ^d^	11.1 (±2.5)^a^, ^d^	11.8 (±2.6)^a^, ^d^	3 (1‐8)^a^, ^b^, ^c^	<.001
Weight (kg)	9 (7.2‐23.8)^c^, ^d^	9.5 (±4.1)^d^	7.5 (2.9‐31.9)^a^, ^d^	27.6 (10‐50)^a^, ^b^, ^c^	<.001
Medications					
Number of dog receiving Spironolactone			17/48		
Spironolactone (mg/kg/d)			2 (1.2‐5.4)		
Number of dog receiving Pimobendan		18/27	48/48		
Pimobendan (mg/kg/d)		0.5 (0.4‐0.7)	0.6 (0.3‐1)		
Number of dog receiving Enalapril			2/48		
Enalapril (mg/kg/d)			0.56 (0.32‐0.8)		
Number of dog receiving Benazepril			37/48		
Benazepril (mg/kg/d)			0.61 (±0.3)		
Number of dog receiving Furosemide			42/48		
Furosemide (mg/kg/d)			4.5 (±1.9)		
Number of dog receiving Torasemide			6/48		
Torasemide (mg/kg/d)			0.4 (±0.1)		
Number of dog receiving Digoxin			4/48		
Digoxin (mg/kg/d)			0.007 (0.006‐0.008)		

*Note*: Data are reported as median and range (minimum‐maximum value) or mean ± SD, based on their distribution.

Abbreviations: Group B1, dogs with myxomatous mitral valve disease in American College of Veterinary Internal Medicine stage B1; group B2, dogs with myxomatous mitral valve disease in American College of Veterinary Internal Medicine stage B2; group C + D, dogs with myxomatous mitral valve disease in American College of Veterinary Internal Medicine stages C and D.

^a^ Significantly different from group B1.

^b^ Significantly different from group B2.

^c^ Significantly different from group C + D.

^d^ Significantly different from healthy dogs.

At the time of enrollment, no dog in group B1 had received cardiovascular drugs. Pimobendan represented the only cardiovascular drug administrated in dogs in group B2; all dogs in group C + D 48/48 (100%) received pimobendan and diuretics. Additionally, 39/48 (81%) dogs of this group received an ACEI and 17/48 (35%) dogs received spironolactone. Among dogs in group C + D, 4/48 (8%) received digoxin to treat atrial fibrillation (Table 1). No other antiarrhythmic drugs were prescribed because no hemodynamically relevant cardiac rhythm disturbances were found in the study population.

Forty‐six healthy dogs were included as controls. The median age was 3 years (range, 1‐8) and the median body weight was 27.6 kg (range, 10‐50). Sex distribution was as follows: 28/46 (61%) were females (16/28 spayed), and 18/46 (39%) were males (7/18 castrated). Thirteen of 46 (28%) were mixed breed dogs whereas 33/46 (72%) were purebred dogs. Healthy dogs were younger and had a higher body weight compared to MMVD dogs (*P* < .001 and *P* < .001, respectively). No difference in sex distribution was documented between healthy and MMVD dogs (*P* = .07).

### 3.2 Clinicopathological data

Among enrolled dogs, 28/98 (27%) were azotemic (sCr between 1.41 and 2.76 mg/dL) whereas 8/48 (17%) had UPC >0.5. Dogs included in group C + D had significantly higher sCr concentration vs B1 and B2 (overall *P* < .001). The UPC was significantly lower in group B1 dogs compared to dogs in both groups B2 and C + D (overall *P* = .002). Urea concentration was significantly higher in group C + D vs groups B1 and B2 and significantly higher in group B2 vs group B1 (overall *P* < .001). In addition, dogs in group C + D had significantly lower serum chloride concentration compared to dogs in group B1 and B2 (overall *P* < .001). No statistical difference was observed between MMVD dogs in different ACVIM stages for leukocyte count and serum CRP concentration (overall *P* = .47 and *P* = .21, respectively). Complete clinicopathological results are reported in Table 2.

**TABLE 2:** Data comparison between dogs with myxomatous mitral valve disease (MMVD) grouped according to their ACVIM stage (group B1, group B2, group C + D)

Variable	RI	Group B1 (n = 23)	Group B2 (n = 27)	Group C + D (n = 48)	*P* value
Creatinine (mg/dL)	0.75‐1.4	0.97 (±0.2)^c^	0.9 (0.5‐2.7)^c^	1.36 (±0.4)^a^, ^b^	<.001
CRP (mg/dL)	0‐1	0.99 (0.5‐4)	0.93 (0.7‐19.7)	1.27 (0.2‐6.4)	.21
Leukocytes (/μL)	6000‐17 000	6485 (5880‐7090)	6970 (4290‐9470)	8815 (5600‐13 700)	.47
Urea (mg/dL)	17‐48	33 (±8.2)^b^, ^c^	42.1 (16‐124)^a^, ^c^	80 (20‐296)^a^, ^b^	<.001
Albumin (g/dL)	2.75‐3.85	3.2 (± 0.19)	3.1 (± 0.32)	3.3 (± 0.4)	.07
Total protein (g/dL)	5.6‐7.30	6.3 (±0.3)^c^	6.3 (±0.6)^c^	6.6 (±0.6)^a^, ^b^	.03
Total calcium (mg/dL)	9.3‐11	10.3 (9.2‐12.2)^c^	9.9 (±0.65)^c^	10.4 (±0.61)^a^, ^b^	.04
Sodium (mEq/L)	143‐151	148 (±1.9)	147 (139‐161)	148 (±3)	.47
Potassium (mEq/L)	3.8‐5.0	4.5 (±0.2)^b^	4.6 (±0.36)^a^, ^c^	4.3 (±0.48)^b^	.004
Chloride (mEq/L)	108.0‐118	111 (±2.68)^c^	111 (±4.3)^c^	106.7 (±3.6)^a^, ^b^	<.001
Magnesium (mg/dL)	1.70‐2.35	2.03 (1.36‐2.3)	2.05 (±0.2)	2.01 (±0.3)	.65
Phosphate (mg/dL)	2.65‐5.40	3.8 (±0.7)	3.9 (±0.9)	3.9 (±1.09)	.62
USG	>1.030	1.030 (±11.6)^c^	1.034 (±12.1)^c^	1.016 (1008‐1065)^a^, ^b^	<.001
UPC (mg/mg)	0‐0.5	0.11 (0.07‐0.8)^b^, ^c^	0.17 (0.07‐0.93)^a^	0.22 (0.05‐1.36)^a^	.002

*Note*: Data are reported as mean ± SD or median and range (minimum‐maximum value), based on their distribution.

Abbreviations: CRP, C‐reactive proteins; group B1, dogs with myxomatous mitral valve disease in American College of Veterinary Internal Medicine stage B1; group B2, dogs with myxomatous mitral valve disease in American College of Veterinary Internal Medicine stage B2; group C + D, dogs with myxomatous mitral valve disease in American College of Veterinary Internal Medicine stages C and D; RI, reference interval; UPC, urine protein to urine creatinine ratio; USG, urine specific gravity.

^a^ Significantly different from group B1.

^b^ Significantly different from group B2.

^c^ Significantly different from group C + D.

### 3.3 Urinary NGAL evaluation in healthy and MMVD dogs

The RI for uNGAL and uNGALC obtained in healthy dogs was 0‐2300 pg/mL and 0‐1400 pg/mg, respectively. No correlation was identified between either uNGAL or uNGALC and age in healthy dogs (*r* = −.02, *P* = .17*; r* = − .01, *P* = .46, respectively). Similarly, no difference in uNGAL and uNGALC results was documented based on sex distribution (*P* = .23, *P* = .07, respectively).

**FIGURE 1:**
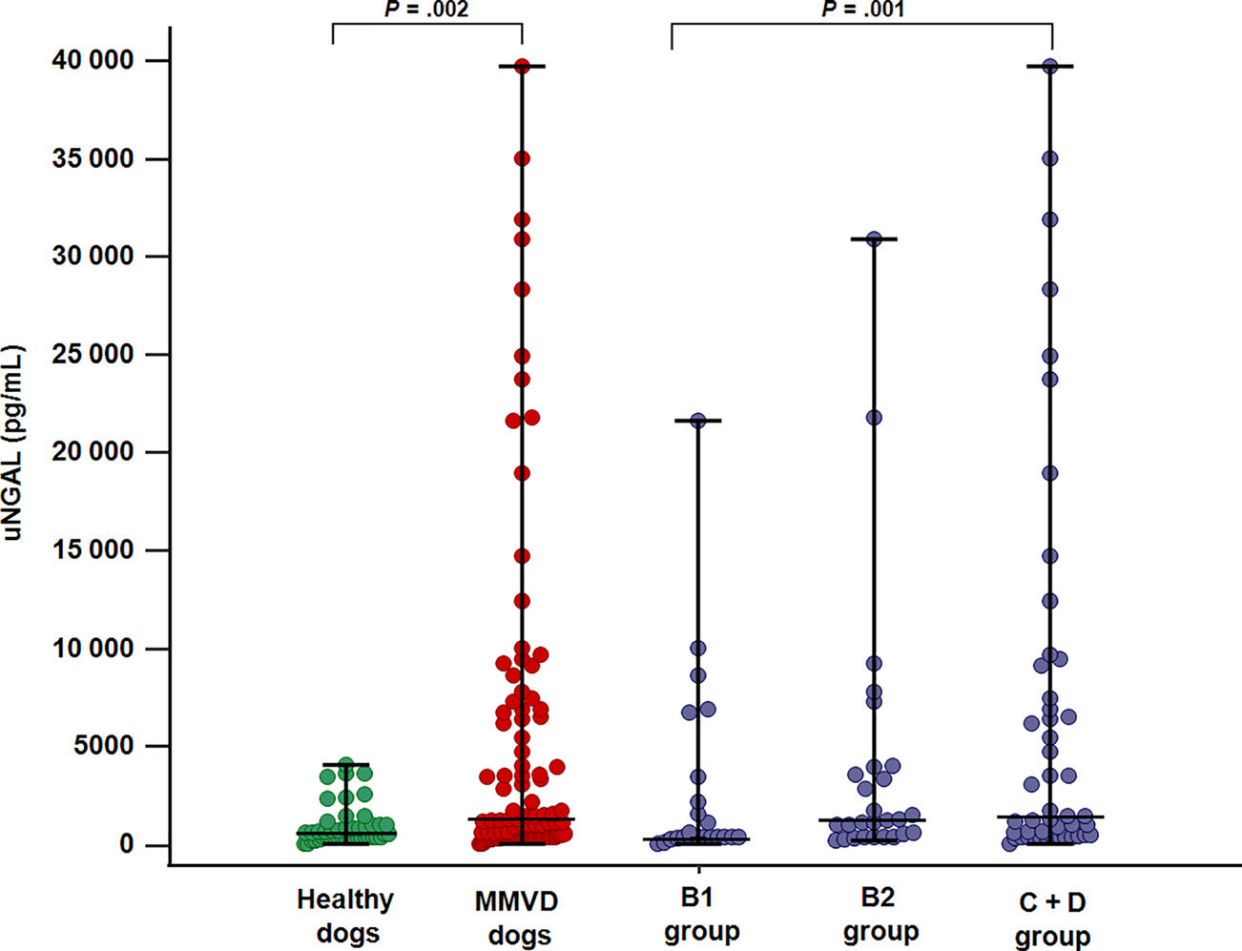
Dot plot showing results of urinary Neutrophil Gelatinase‐Associated Lipocalin (uNGAL) comparison between healthy dogs (n = 46) (green dots) and dogs with myxomatous mitral valve disease (MMVD) (n = 98) (red dots), and among dogs with MMVD in different ACVIM stages (blue dots): group B1 (n = 23), group B2 (n = 27) and group C + D (n = 48). Upright bars represent minimum and maximum values, while horizontal lines (central bars) represent median value. P values are reported for significantly different results (*P* < .05). uNGAL: urinary Neutrophil Gelatinase‐Associated Lipocalin; MMVD dogs: dogs with myxomatous mitral valve disease; B1 group: dogs with myxomatous mitral valve disease in ACVIM stage B1; B2 group: dogs with myxomatous mitral valve disease in ACVIM stage B2; C + D group: dogs with myxomatous mitral valve disease in ACVIM stage C and D

**FIGURE 2:**
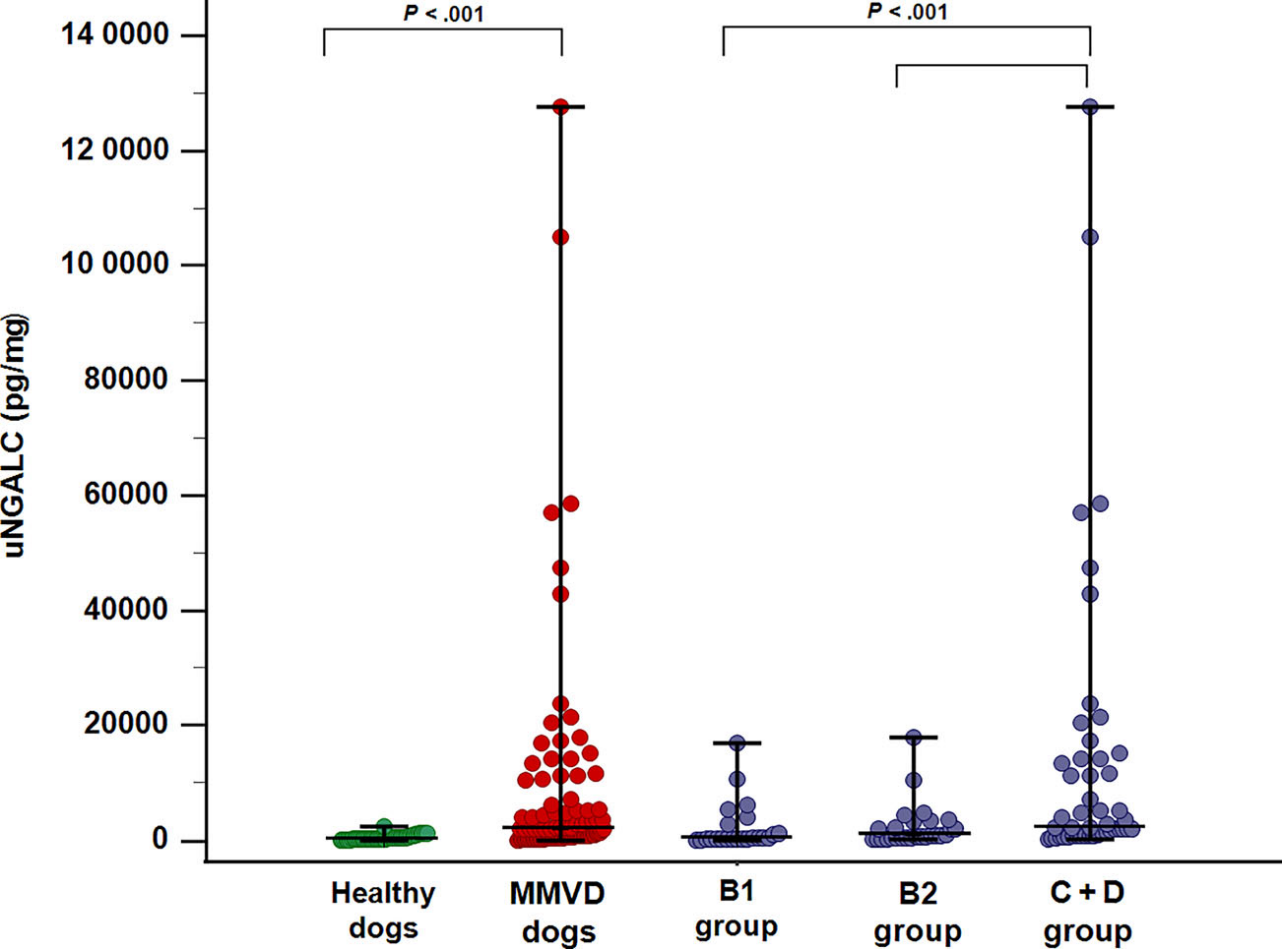
Dot plot showing results of urinary Neutrophil Gelatinase‐Associated Lipocalin to urinary creatinine ratio (uNGALC) comparison between healthy dogs (n = 46) (green dots) and dogs with myxomatous mitral valve disease (MMVD) (n = 98) (red dots), and among dogs with MMVD in different ACVIM stages (blue dots): group B1 (n = 23), group B2 (n = 27) and group C + D (n = 48). Upright bars represent minimum and maximum values, while horizontal lines (central bars) represent median value. *P* values are reported for significantly different results (*P* < .05). uNGALC: urinary Neutrophil Gelatinase‐Associated Lipocalin to urinary creatinine ratio; MMVD dogs: dogs with myxomatous mitral valve disease; B1 group: dogs with myxomatous mitral valve disease in ACVIM stage B1; B2 group: dogs with myxomatous mitral valve disease in ACVIM stage B2; C + D group: dogs with myxomatous mitral valve disease in ACVIM stage C and D

In the comparison of healthy and MMVD dogs, the overall population of dogs with MMVD had significantly increased uNGAL (1204 pg/mL; range, 30‐39 732) and uNGALC (1816 pg/mg; range, 22‐127 693) compared to healthy dogs (uNGAL, 584 pg/mL; range, 56‐4072; uNGALC, 231 pg/mg; range, 15‐2407; *P* = .002, *P* < .001, respectively; Figures 1 and 2).

When uNGAL values were compared among MMVD dogs in different ACVIM stages, dogs belonging to group C + D had significantly higher uNGAL compared to group B1 and healthy dogs. Furthermore, uNGAL was significantly higher in group B2 compared to healthy controls, whereas no difference was detected in the comparison between group B1 vs B2, between group B2 vs group C + D and between B1 vs healthy dogs (overall *P* = .001). Concerning uNGALC results, all dogs with MMVD had higher values compared to healthy controls, with dogs included in group C + D having significantly higher uNGALC compared to the other MMVD groups (overall *P* < .001; Table 3; Figures 1 and 2).

**TABLE 3:** uNGAL, uNGALC, and uCr comparison between dogs with myxomatous mitral valve disease (MMVD) grouped according to their ACVIM stage (group B1, group B2, group C + D), and between healthy control dogs

Variable	Group B1 (n = 23)	Group B2 (n = 27)	Group C + D (n = 48)	Healthy dogs (n = 46)	*P* value
uCr (mg/dL)	183 (±80)^c^, ^d^	171 (±62)^c^, ^d^	50 (14‐226)^a^, ^b^, ^d^	297 (±129.7)^a^, ^b^, ^c^	<.001
uNGAL (pg/mL)	400 (70‐21 647)^c^	1231 (208‐30 916)^d^	1463 (30‐39 732)^a^, ^d^	584 (56‐4072)^b^, ^c^	.001
uNGALC (pg/mg)	304 (22‐16 871)^c^, ^d^	777 (109‐17 989)^c^, ^d^	2478 (177‐127 639)^a^, ^b^, ^d^	231 (15‐2407)^a^, ^b^, ^c^	<.001

*Note*: Data are reported as mean ± SD or median and range (minimum‐maximum value), based on their distribution.

Abbreviations: uCr, urinary creatinine; uNGAL, urinary neutrophil gelatinase‐associated lipocalin; uNGALC, urinary neutrophil gelatinase‐associated lipocalin to urinary creatinine ratio; group B1, dogs with myxomatous mitral valve disease in American College of Veterinary Internal Medicine stage B1; group B2, dogs with myxomatous mitral valve disease in American College of Veterinary Internal Medicine stage B2; group C + D, dogs with myxomatous mitral valve disease in American College of Veterinary Internal Medicine stage C and D.

^a^ Significantly different from group B1.

^b^ Significantly different from group B2.

^c^ Significantly different from group C + D.

^d^ Significantly different from healthy dogs.

In dogs with MMVD, frequency of abnormal uNGAL results (ie, values above the RI) increased with increasing ACVIM stage: 26% (6/23) in dogs in group B1, 37% (10/27) in dogs in group B2, and 46% (22/48) in dogs in group C + D (*P* = .27). A similar and more significant trend was noted for uNGALC results, because these were above the RI in 26% (6/23) of dogs in group B1, 41% (11/27) of dogs in group B2, and 73% (35/48) of dogs in group C + D (*P* = .0003).

A significant positive correlation was documented for both uNGAL and uNGALC with UPC (*r* = .47, *P* < .001*; r* = .54, *P* < .001, respectively) and urea (*r* = .27, *P* = .0071; *r* = .45, *P* < .001, respectively). Serum creatinine and CRP concentrations and leukocyte count were not significantly correlated with uNGAL (*r* = .02, *P* = .08; *r* = .14, *P* = .16; *r* = .14, *P* = .59, respectively) and uNGALC (*r* = .15, *P* = .12*; r* = .15, *P* = .14; *r* = −.07, *P* = .78, respectively). Moreover, no correlation was found between uNGAL and uNGALC with daily furosemide dosage (*r* = .005, *P* = .97*; r* = .09, *P* = .53, respectively). No correlation was performed for torasemide given the low number of dogs receiving this drug.

## 4 DISCUSSION

Our study aimed to assess the presence of renal tubular damage in dogs with stable MMVD and to evaluate changes according to the severity of heart disease. The deterioration of renal function linked to chronic cardiac disease by both chronological and causal relationship (so‐called CRS type II) has been a matter of growing debate in human medicine, but only scarcely characterized in veterinary medicine.^3^, ^5^, ^6^, ^7^, ^17^ In our opinion, such a gap of knowledge can have relevant clinical implications, because renal damage and tubular injury could affect prognosis, influence therapeutic decision‐making and impair response to treatment.^4^, ^5^ For the purpose of our study, we enrolled dogs with MMVD, a disease with a high prevalence and a chronic and progressive course, sometimes requiring life‐long diuretic treatment,^1^, ^2^ representing an excellent research model to fill the knowledge gap. In accordance with our initial hypothesis, uNGAL is increased in dogs with MMVD compared to healthy controls and increases with worsening cardiac disease. This finding suggests the presence of subclinical tubular damage in all MMVD stages, occurring in the absence of clinical signs of renal involvement and severe azotemia in the majority of the cases. Indeed, the values of uNGAL documented in enrolled MMVD dogs are far lower compared to those reported in dogs with AKI,^13^ but still abnormal with respect to the healthy state.

In humans, serum and uNGAL are among the most studied biomarkers of tubular damage in AKI, and increases have been documented extensively in patients with CRS caused by different cardiovascular diseases, including those leading to CHF.^16^ For example, a previous study showed that renal impairment in patients with CHF with stable disease is not only characterized by decreased glomerular filtration rate, but also by higher uNGAL results compared to control subjects, indicating the presence of renal tubular damage.^21^ Interestingly, another study documented that serum NGAL represents a marker of renal injury in people with CHF even when sCr is within the RI.^22^ In humans, serum and uNGAL also correlate with the clinical severity of CHF and, occasionally, with indices of ventricular structural damage and dysfunction.^16^, ^23^ In dogs, recent studies determined that serum and uNGAL act as a sensitive and specific biomarkers of AKI and tubular injury, despite being subjected to potential influence during systemic inflammation.^13^, ^24^, ^25^ However, as previously mentioned, the literature concerning NGAL as a biomarker of CRS is currently scarce in dogs. A recent study identified higher serum NGAL in dogs with acute CHF caused by MMVD compared to healthy dogs. Moreover, higher serum NGAL concentrations were noticed upon admission in dogs with CHF that developed worsening of renal function within 7 days of hospitalization compared to those with stable sCr, thus highlighting the potential role of NGAL as an early biomarker of AKI during acute CHF.^17^ To our knowledge, tubular damage in dogs with stable MMVD has never been extensively evaluated. A preliminary evaluation of some biomarkers of kidney damage (clusterin, cystatin B, inosine and NGAL) has been reported previously.^3^ In that study, the hypothesized mechanisms contributing to increases in those biomarkers were episodes of intermittent AKI or sustained kidney injury occurring simultaneously with a progressive reduction of functional kidney mass.^3^ In that same study, despite being cited, uNGAL results were not reported. Based on our findings, progressive and sustained kidney damage could represent the main mechanism behind CRS type II in MMVD dogs with stable disease, even if the role of episodic AKI cannot be completely ruled out.

The normal function of the cardiorenal axis contributes to normal cardiovascular homeostasis.^7^ During MMVD and CRS type II, different scenarios are possible ranging from a chronic decrease in effective circulating volume, with subsequent renal hypoperfusion and ischemia because of decreased cardiac output, as well as chronic renal congestion and venous hypertension causing impaired intrarenal blood flow.^7^, ^26^ In this regard, the renin‐angiotensin‐aldosterone system (RAAS) and neurohormonal activation have been associated with fluid overload, venous hypertension, and renal interstitial edema in humans and in canine experimental models of disease, and have been implicated in CKD progression because of ongoing renal interstitial fibrosis and glomerulosclerosis in humans and in animal models of CRS type II.^7^, ^26^, ^27^, ^28^ Moreover, episodic cardiac events and repeated occurrence of AKI induce progressive hypoxic and ischemic insults to the kidneys.^7^, ^29^ These events, overall, can be expressed at any stage of cardiac disease, and become progressively more evident with worsening heart failure.^3^ All of these mechanisms can justify the progressive increase in uNGAL in our study population with increasing ACVIM CHF stages as well as the higher prevalence of abnormal uNGAL in the more advanced stages of MMVD.

In this regard, diuretic treatment might play a role. Indeed, diuretics induce subclinical changes in volume status, which might affect renal and tubular function and integrity. Moreover, because furosemide acts at the tubular level, it theoretically might affect renal excretion of NGAL. However, this effect has not been identified in recent studies of humans.^30^, ^31^ For example, in 1 study, although diuretic withdrawal was associated with an increase in some biomarkers of tubular dysfunction, which returned to within normal ranges after diuretic reinstitution, uNGAL concentrations were unaffected by changes in diuretic treatment.^32^ Similar data are lacking in the veterinary literature. In our study, no significant difference in uNGAL results was found between ACVIM stage C and D dogs when considering these stages as separate entities (data not shown), and no correlation was detected between uNGAL and furosemide dosage. In our opinion, a relevant role of diuretic treatment in the excretion of NGAL is unlikely but this hypothesis should be confirmed in additional studies designed with this particular aim.

Another possibility for increases in uNGAL in enrolled MMVD dogs could be the presence of preexisting CKD. Previous studies documented higher uNGAL in dogs with CKD compared to healthy dogs,^20^ and uNGAL seems to predict the risk of progressive vs stable CKD in dogs.^33^ Both CKD and chronic valvular disease usually occur in elderly patients, and the estimated prevalence of CKD seems higher in dogs with MMVD than in the general canine population.^4^ In our opinion, the possibility of preexisting CKD influencing uNGAL in the dogs enrolled in our study seems unlikely. Indeed, uNGAL was above the RI in approximately half of the study population of MMVD dogs (uNGAL, 38/98, 39%; uNGALC, 52/98, 53%) and, although not directly comparable, the uNGAL results that we documented seem overall lower compared those reported in dogs with CKD.^20^, ^33^ In any case, preexisting renal disease causing the increase in uNGAL would be active progressive renal damage that worsens with increasing ACVIM stage and hence still would fall into CRS type II.

Among the potential nonrenal causes of increased NGAL in cardiac disease, chronic inflammation and cardiac remodeling should be mentioned. In humans, chronic inflammation is a hallmark of CHF, and inflammatory mediators have been implicated in cardiovascular disease progression.^7^, ^34^ Systemic inflammation could cause an increase in circulating NGAL concentration because NGAL is produced by leukocytes. The role of inflammation in uNGAL excretion, however, is not well defined in humans with heart disease.^16^ According to a previous study, higher uNGAL was reported in dogs with inflammatory AKI compared to dogs with noninflammatory AKI.^13^ Similar to what has been reported in human medicine, mild systemic inflammation has been reported in cardiac diseases of dogs,^35^, ^36^, ^37^ and may contribute, at least in part, to increased NGAL. In our study population, however, the leukocyte count was normal, serum CRP concentrations were only mildly increased, and these results were similar in MMVD dogs of different ACVIM stages. In addition, no correlation was detected between both leukocyte count and serum CRP with uNGAL, making an effect of systemic inflammation on our results unlikely.

In human patients with cardiac diseases, NGAL also plays a direct role in the pathogenesis of cardiovascular remodeling as well as in atherosclerotic plaque instability.^7^, ^38^ Moreover, NGAL upregulation has been demonstrated in the myocardial tissue of human patients who died of CHF.^7^, ^38^ Similarly, in a rat model of postmyocardial infarction heart failure, NGAL/lipocalin‐2 gene expression was found to be increased in cardiomyocytes in both normal and failing myocardium.^8^ Because cardiac remodeling occurs in dogs along with progression of MMVD,^1^ cardiac expression of NGAL also may be hypothesized in this species, but has not been documented in dogs so far. Based on our study design, the contribution of cardiac remodeling to NGAL results in our population is hard to evaluate, but remains a possibility that should be clarified in future studies.

Besides higher uNGAL, dogs with ACVIM stage C + D MMVD also had higher median concentrations of urea, sCr and higher UPC compared to MMVD dogs with less severe disease. More severe impairment of both glomerular and tubular function seems to occur in the more advanced stages of MMVD in dogs, likely because of more severe renal hypoperfusion associated with vigorous diuretic treatment,^4^ or because of frequent renal hypoxic insults associated with worsening heart disease and decompensation.^3^ Moreover, these dogs could experience progressive kidney damage leading to renal fibrosis and CKD, as previously noted and already discussed.^3^, ^4^, ^39^ Interestingly, no correlation was found between uNGAL and sCr in our study population. This result, however, is expected, because NGAL indicates renal tubular damage whereas sCr is a surrogate of renal function impairment. Specifically, in the context of heart failure, a combined approach measuring both sCr and uNGAL might be preferred to correctly assess renal damage and dysfunction. As an example, iatrogenic prerenal azotemia caused by overzealous diuretic treatment might not necessarily cause parenchymal damage, whereas small decreases in renal function might result in tubular hypoxia and subsequent tubular damage despite relatively normal glomerular filtration rate and sCr.^17^ Finally, uNGAL can reflect subclinical injury and could anticipate an increase in sCr. With regard to UPC, most of the MMVD dogs in our study were nonproteinuric or borderline proteinuric according to the IRIS guidelines for CKD.^40^ Interestingly, such a finding is consistent with previous studies evaluating renal compromise in dogs with MMVD.^39^, ^41^ Low‐grade proteinuria can be associated with tubular damage, and is consistent with the mild positive correlation observed in our study between uNGAL and UPC. Nonetheless, better characterization of proteinuria in dogs with MMVD using qualitative methods would be needed to confirm its tubular origin.

Our study had some limitations. The main limitation was that dogs with MMVD and control dogs were not matched for age and body weight. Specifically, healthy control dogs were younger than dogs with MMVD. Urinary NGAL in people is linearly related to aging. Such variations are mild, potentially related to loss of renal mass and tubulointerstitial fibrosis, but enough to consider the establishment of age‐related reference values in the healthy population.^42^ Indeed, both cardiovascular and renal disease are considered age‐related diseases in people, making it difficult to evaluate the effect of age on a specific biomarker separately from age‐associated comorbidities. Whether such age‐associated effects on urinary biomarkers are clinically relevant remains a matter of debate in humans. To the best of our knowledge, no similar data are available in dogs. Although an age effect was not identified in healthy dogs enrolled in our study, the possible impact of aging on uNGAL results should be acknowledged in MMVD dogs. In the included MMVD dogs, age increased with increasing ACVIM stage (data not shown). This finding might be partially expected, because MMVD could progress with aging. Aging also could be a predisposing factor for progressive functional renal loss and CKD, which could be an additional reason for the obtained NGAL results, as previously discussed. Nonetheless, it seems unlikely that aging alone would be a determining factor behind the increase in uNGAL identified in our study population. However, because of the difficulty in overcoming this limitation when evaluating diseased animals, additional studies specifically addressing the age effect on canine uNGAL in healthy dogs are needed. Because of the low number of ACVIM stage D dogs, they were incorporated with stage C cases (group C + D) for statistical purposes. This choice might have caused some heterogeneity because of different disease severities and diuretic needs. In addition, no tissue samples to evaluate for the presence of structural kidney damage consistent with CRS type II were collected in enrolled dogs. Moreover, additional tests to characterize tubular damage (eg, urine protein electrophoresis, other renal biomarkers) and to assess venous congestion (eg, abdominal ultrasound) could have been useful to better explore uNGAL origin and correlations in our study. Although dogs with signs of urinary tract infection or pyuria were excluded from our study, urine cultures were not systematically performed, which could be considered a minor limitation. Lastly, although the number of control dogs we enrolled is substantially higher than in previous studies in dogs that evaluated renal impairment in MMVD,^39^, ^41^ we did not achieve the sample size recommended by the American Society of Veterinary Clinical Pathology when defining RIs (ie, ≥120 subjects).^43^ For this reason, further refinement of RIs should be carried out in the future.

In conclusion, our study identified the presence of renal tubular damage in dogs with stable MMVD. This tubular damage was subclinical, and evident even in the initial stages of the disease in dogs not receiving diuretic treatment. This finding emphasizes that MMVD dogs experience functional kidney impairment beyond that related to hemodynamic changes associated with cardiac disease and diuretics. Increasing uNGAL along with the worsening of heart disease indicates that renal damage during MMVD in dogs might be progressive and potentially involved in renal fibrosis, renal aging, and CKD development in more advanced MMVD stages. The role of reno‐protective approaches in the management of dogs with MMVD should be explored in the future because they can potentially slow progression and decrease complications of CRS.

## CONFLICT OF INTEREST DECLARATION

Authors declare no conflict of interest.

## OFF‐LABEL ANTIMICROBIAL DECLARATION

Authors declare no off‐label use of antimicrobials.

## INSTITUTIONAL ANIMAL CARE AND USE COMMITTEE (IACUC) OR OTHER APPROVAL DECLARATION

Approved by Scientific Ethical Committee for Animal Testing of the Alma Mater Studiorum.

## HUMAN ETHICS APPROVAL DECLARATION

Authors declare human ethics approval was not needed for this study.
